# Supplementary material for: Single-cell transcriptomic analysis of bloodstream Trypanosoma brucei reconstructs cell cycle progression and developmental quorum sensing
Source: Nat Commun. 2021 Sep 6;12:5268. doi: 10.1038/s41467-021-25607-2 (PMC8421343; doi:10.1038/s41467-021-25607-2)
Supplement: Supplementary file 1 — Supplementary Information [file 41467_2021_25607_MOESM1_ESM.pdf]

SUPPLEMENTARY INFORMATION

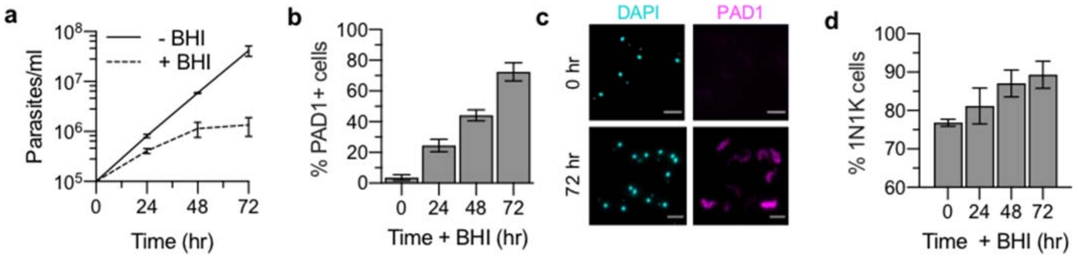

Figure S1. Brain heat infusion (BHI) broth induces slender to stumpy differentiation *in vitro.* **a**) Cumulative growth of pleomorphic *T. brucei* in culture with (dashed line) and without (solid line) 10% BHI broth. y-axis shows parasites per ml on a log10 scale. Data is presented as the mean +/- SD of three independent replicates. **b**) Percentage of cells expressing stumpy marker protein PAD1 after culturing with 10% BHI. Data is presented as the mean +/- SD of three independent replicates. **c**) Staining of parasites with anti-PAD1 antibody, after 72 hr incubation with 10% BHI broth. Scale, 5 μm. **d**) Percentage of 1N1K *T. brucei* after culturing with 10% BHI broth. Data is presented as the mean +/- SD of three independent replicates.

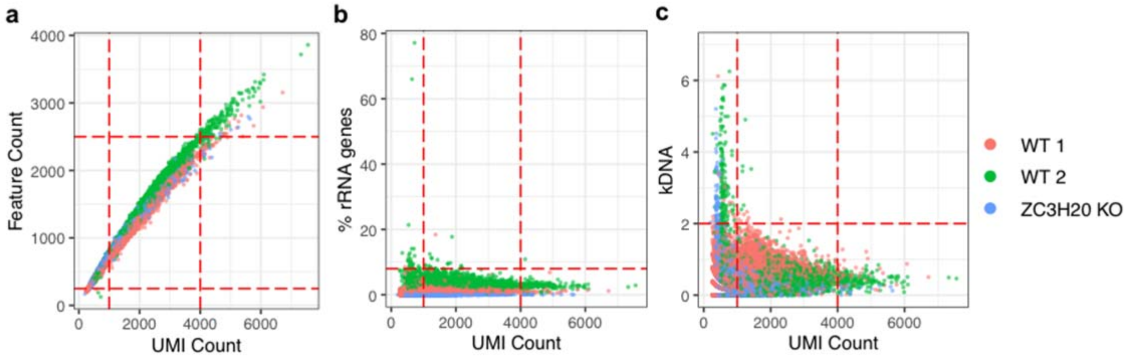

Figure S2. Quality control and filtering of single transcriptomes. Scatter plots for WT replicate 1 (red) and 2 (green), ZC3H20 KO (blue) experiments; each data point is one transcriptome. Plots show the relationship between the number of unique molecular identifiers detected per cell and (**a**) number of features (genes), (**b**) percentage of features encoding ribosomal RNA (rRNA) and (**c**) percentage of features encoding on the kDNA maxi circle genome. Red dashed lines indicate thresholds used to filter cells per experiment; Features count > 250, < 2500, UMI count > 1000, < 4000, % rRNA genes < 8, % kDNA genes < 2.

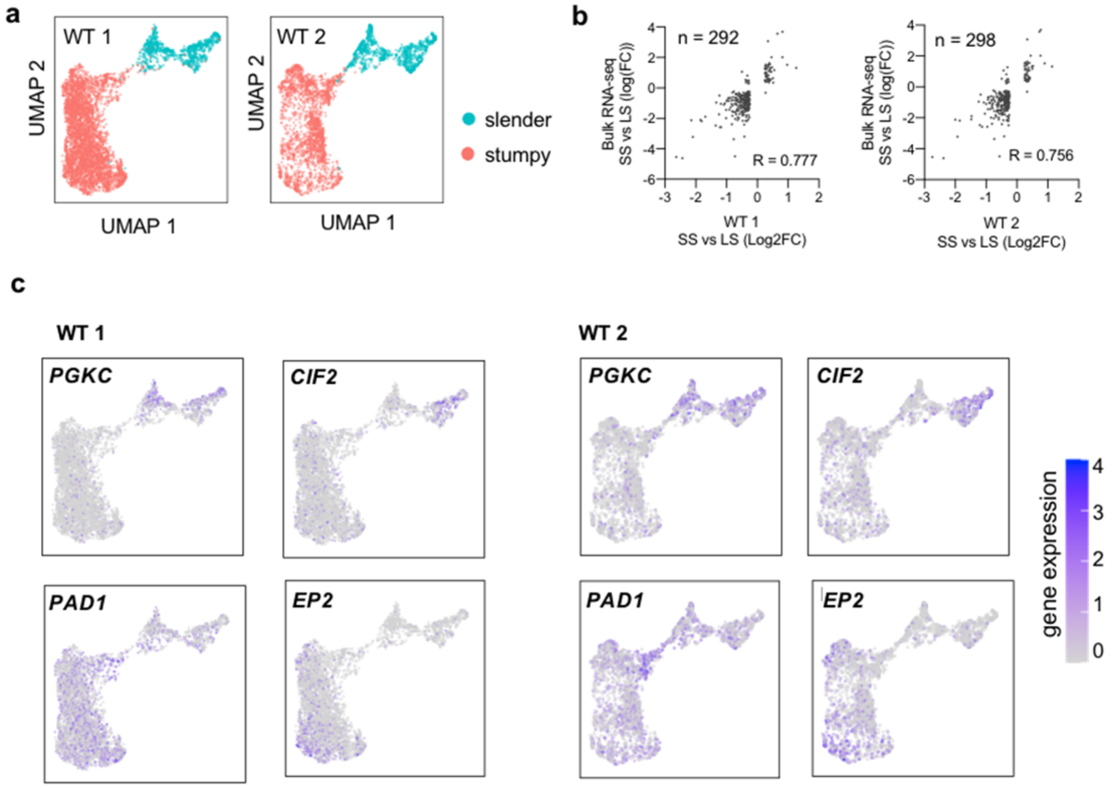

Figure S3. Comparison of scRNA-seq and bulk transcriptomic analysis of *in vivo* slender and stumpy forms. a) UMAP plots of slender- (blue) and stumpy-like (red) cells for each WT replicate experiment. **b**) Log2(fold-change) in transcript levels between stumpy and slender cells captured by scRNA-seq (x-axis) and stumpy and slender rich populations analysed by bulk RNA-seq (y-axis) for two replicate experiments (WT 1, left and WT 2, right). Correlation was analysed using two-tailed Pearson R test, indicated for each replicate. 'n' indicates the number of genes significantly (adjusted p value <0.05) differentially expressed in each scRNA-seq replicate and bulk-RNA analysis. **c**) UMAP of WT parasites for experimental replicates 1 (left) and 2 (right), coloured by transcript counts for two slender marker genes (PGKC; Tb927. 1.700 and CIF2; Tb927. 9.14290) and two stumpy marker genes (PAD1; Tb927. 7.5930 and EP2; Tb927. 10.10250). Scale shows raw transcript count per cell.

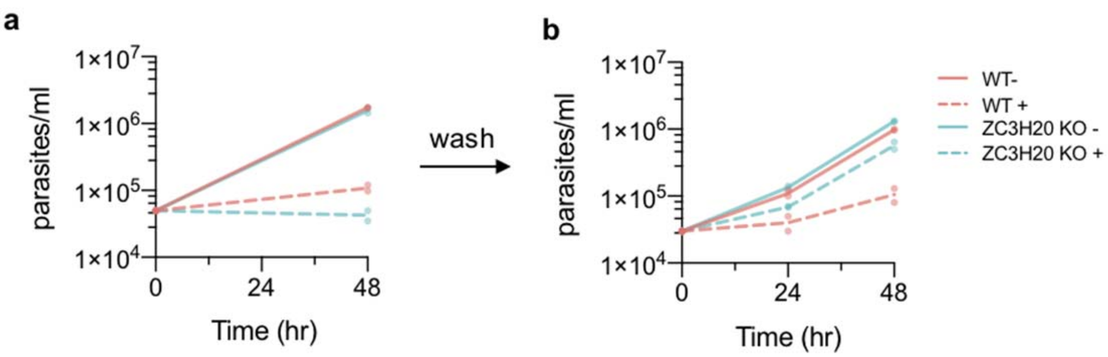

Figure S4. Recovery of WT and ZC3H20 KO *T. brucei* after BHI-induction of differentiation. **a)** Growth curve of ZC3H20 KO (red) and parental WT line (containing Cas9 and T7 polymerase expression plasmid; blue) with (dashed lines) and without (solid lines) 10% BHI. y-axis shows parasites per ml on a log10 scale. Use of a different fresh batch of BHI has caused a more pronounced growth defect here than in Fig. 4a. **b**) Growth curve of parasites after washing to remove BHI and recovered in fresh media. In both cases the mean of two independent replicates is plotted, error bars plot SD from the mean.

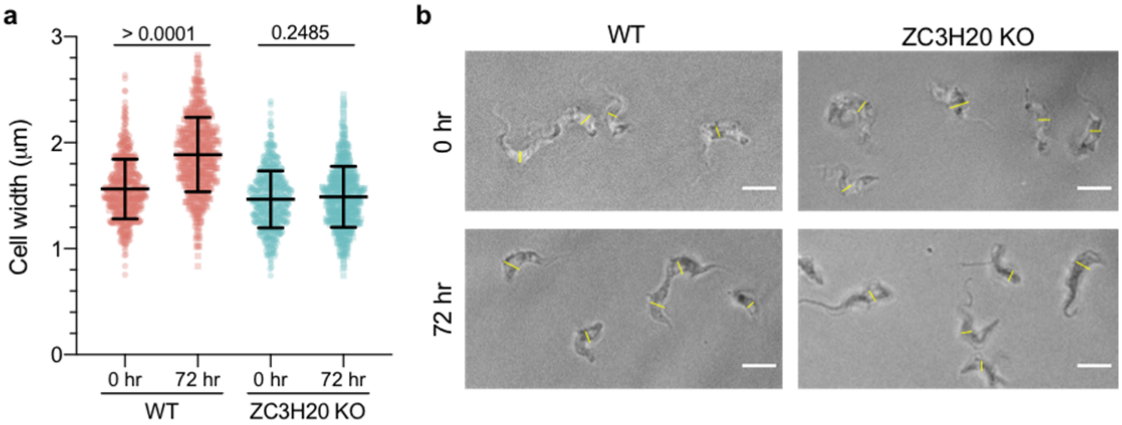

Figure S5. Change in cell morphology of WT and ZC3H20 KO *T. brucei* after BHI-induction of differentiation a) Width of WT (red) and ZC3H20 KO (blue) *T. brucei* parasites (µm) before incubation with 10% BHI (circles) and after 72 hr of culture with BHI (squares). Line shows the mean of at least 572 parasites from three independent experiments (at least 177 from each replicate). Error bars show the standard deviation of all points. Two-tailed Mann Whitney test was performed between 0 and 72 hr time points for WT and ZC3H20 KO parasites separately, *p*-values are indicated above. **b)** Example DIC images of WT and ZC3H20 KO *T. brucei* before and 72 hr after BHI treatment. Yellow lines indicate how cell width was measured. White scale bars, 5 µm.

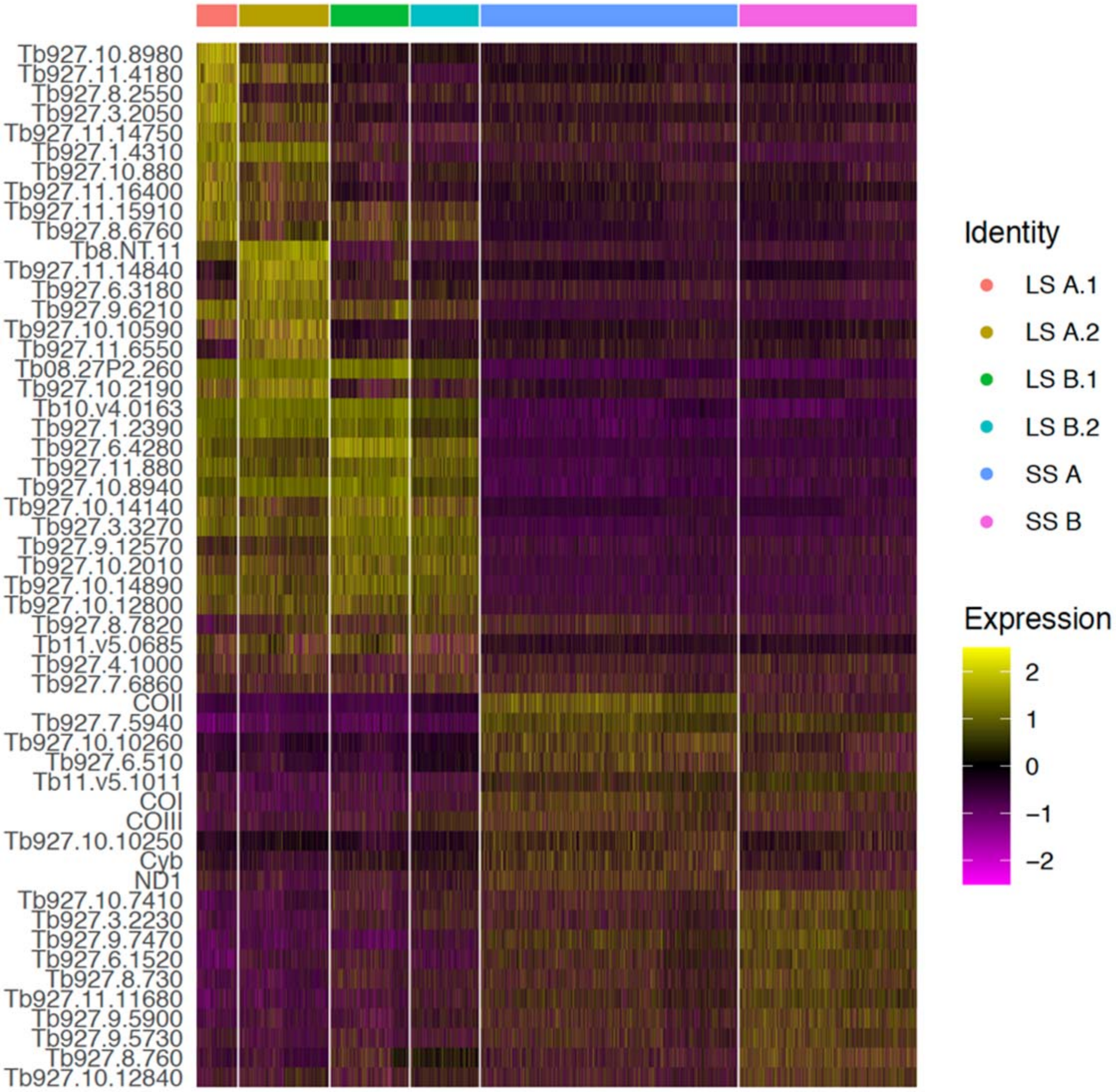

Figure S6. WT and ZC3H20 KO cluster maker genes. Heatmap showing relative expression levels (log2 normalised z-score) of the top 10 maker genes of each cluster identified in **5d**. Each row is one gene coloured by relative expression. The gene ID is given for each marker. Each column is one cell grouped according to cluster identity.
