# Supplementary material for: Single-cell transcriptomic analysis of bloodstream Trypanosoma brucei reconstructs cell cycle progression and developmental quorum sensing
Source: Nat Commun. 2021 Sep 6;12:5268. doi: 10.1038/s41467-021-25607-2 (PMC8421343; doi:10.1038/s41467-021-25607-2)
Supplement: Supplementary file 3 — Description of Additional Supplementary Files [file 41467_2021_25607_MOESM3_ESM.pdf]

Description of Additional Supplementary Files

File Name: Supplementary Data 1
Description: scRNA-seq sample metrics, multiplet identification and variable genes.

File Name: Supplementary Data 2
Description: Analysis of WT differentiating T. brucei: cluster marker genes (identified with MAST; adusted p-value < 0.05 and mean log FC > 0.25), associated GO term enrichement (two-tailed Fisher's exact test p-values and Benjamini adjusted p-values), genes differentially expressed during WT differentiation (identified by associationTest; p1193 value < 0.05 and FC > 2) and associated GO term enrichement two-tailed Fisher's exact test p-values and Benjamini adjusted p-values) .

File Name: Supplementary Data 3
Description: Cell cycle analysis of slender form T. brucei: genes differentially expressed during the WT slender cell cycle (identified by associationTest; p-value < 0.05 and FC > 2).

File Name: Supplementary Data 4
Description: Analysis ZC3H20 KO T. brucei: cluster marker genes (identified with MAST; adusted p-value < 0.05 and mean log FC > 0.25), genes differentially expressed during the branched differentiation trajectory of WT and ZC3H20 KO parasites (identified by associationTest; p-value < 0.05 and FC > 2), genes differentially expressed early during differentiation (identified by earlyDETest; p-value < 0.05 and FC > 2).
