# Supplementary material for: Single-cell transcriptomic analysis of bloodstream Trypanosoma brucei reconstructs cell cycle progression and developmental quorum sensing
Source: Nat Commun. 2021 Sep 6;12:5268. doi: 10.1038/s41467-021-25607-2 (PMC8421343; doi:10.1038/s41467-021-25607-2)
Supplement: Supplementary file 8 — Reporting Summary [file 41467_2021_25607_MOESM8_ESM.pdf]

nature research

Corresponding author(s): Emma Marie Briggs

Last updated by author(s): Aug 5, 2021

Reporting Summary

Nature Research wishes to improve the reproducibility of the work that we publish. This form provides structure for consistency and transparency in reporting. For further information on Nature Research policies, see our Editorial Policies and the Editorial Policy Checklist.

Statistics

For all statistical analyses, confirm that the following items are present in the figure legend, table legend, main text, or Methods section.

n/a	Confirmed	
☐	☒	The exact sample size (*n*) for each experimental group/condition, given as a discrete number and unit of measurement
☐	☒	A statement on whether measurements were taken from distinct samples or whether the same sample was measured repeatedly
☐	☒	The statistical test(s) used AND whether they are one- or two-sided<br>*Only common tests should be described solely by name; describe more complex techniques in the Methods section.*
☒	☐	A description of all covariates tested
☐	☒	A description of any assumptions or corrections, such as tests of normality and adjustment for multiple comparisons
☐	☒	A full description of the statistical parameters including central tendency (e.g. means) or other basic estimates (e.g. regression coefficient) AND variation (e.g. standard deviation) or associated estimates of uncertainty (e.g. confidence intervals)
☐	☒	For null hypothesis testing, the test statistic (e.g. *F*, *t*, *r*) with confidence intervals, effect sizes, degrees of freedom and *P* value noted<br>*Give P values as exact values whenever suitable.*
☒	☐	For Bayesian analysis, information on the choice of priors and Markov chain Monte Carlo settings
☒	☐	For hierarchical and complex designs, identification of the appropriate level for tests and full reporting of outcomes
☐	☒	Estimates of effect sizes (e.g. Cohen's *d*, Pearson's *r*), indicating how they were calculated

Our web collection on statistics for biologists contains articles on many of the points above.

Software and code

Policy information about availability of computer code

Data collection	Fiji v2 was used for image analysis.
Data analysis	All software is freely available (R v3.6.3, Seurat v3. 2.2.99, clusterExperiment v2.6.1, MAST vl.12.0, scran 1.14.6, Seurat 3.2.2.99 SingleCellExperiment vl.8.0, slingshot 1.4.0, STACAS vl.0.1, tradeSeq 1.3.21, PhateR 1.0.7, seater 1.14.6, princurve v2.1.4, Cell ranger v3.0.2). All code is available at 10.5281/zenodo.5163554

For manuscripts utilizing custom algorithms or software that are central to the research but not yet described in published literature, software must be made available to editors and reviewers. We strongly encourage code deposition in a community repository (e.g. GitHub). See the Nature Research guidelines for submitting code & software for further information.

Data

Policy information about availability of data

All manuscripts must include a data availability statement. This statement should provide the following information, where applicable:
- Accession codes, unique identifiers, or web links for publicly available datasets
- A list of figures that have associated raw data
- A description of any restrictions on data availability

Data can be sourced via Supplementary Data Tables and the European Nucleotide Archive with accession number PRJEB41744. Processed scrna-seq data can be accessed at 10.5281/zenodo.5163554. Wild-type scRNA-seq data can be explored using the interactive cell atlas (http://cellatlas.mvls.gla.ac.uk/TbruceiBSF/). Raw data associated with figures 1d, 2e, 4a, 4f, 5c, 5f, S1a, S1b, S1c, S3b, S4a, S4b and S5a are provided in Source data file.

April 2020

Field-specific reporting

Please select the one below that is the best fit for your research. If you are not sure, read the appropriate sections before making your selection.

- [x] Life sciences
- [ ] Behavioural & social sciences
- [ ] Ecological, evolutionary & environmental sciences

For a reference copy of the document with all sections, see nature.com/documents/nr-reporting-summary-flat.pdf

Life sciences study design

All studies must disclose on these points even when the disclosure is negative.

Sample size	The maximum sample size was used for each single cell RNA-seq experiment, within the limitations of the technology (15,000 cells per experiment). For other experiments sample no sizes were calculated. All analyses were carried out in independent biological replicates (at least n=2), after pilot analyses. All outcomes were consistent.
Data exclusions	For WT 1, WT 2 and ZC3H20 single cell transcriptomic experiments, cell transcriptomes were excluded from the analysis if they were determined to contain transcripts of multiple cells or from inviable cells.
Replication	All experiments were performed at least biological duplicate and attempts to replicate were successful. Single cell transcriptomics analysis was performed with samples which behaved in the same manner as biological replicates in test conditions; two wild-type biological replicated were examined by scRNA-seq and one ZC3H20 KO replicate.
Randomization	Randomisation scRNA was not appropriate as biological replicates were carried out independently at different times. For cell growth analyses randomisation was not carried out in order to allow inclusion of appropriate culture additives for the respective cell lines. All analyses were replicated and consistent.
Blinding	Blinding of scRNA was not appropriate as data processing and analysis need to be carried out appropriately and differentially for biological wild type replicates vs ZC3H20 KO sample.<br><br>For cell growth and IFA analyses blinding was not carried out in order to allow inclusion of appropriate culture additives for the respective cell lines. All analyses were replicated and consistent

Reporting for specific materials, systems and methods

We require information from authors about some types of materials, experimental systems and methods used in many studies. Here, indicate whether each material, system or method listed is relevant to your study. If you are not sure if a list item applies to your research, read the appropriate section before selecting a response.

Materials & experimental systems

n/a	Involved in the study	
[ ]	[x]	Antibodies
[ ]	[x]	Eukaryotic cell lines
[x]	[ ]	Palaeontology and archaeology
[x]	[ ]	Animals and other organisms
[x]	[ ]	Human research participants
[x]	[ ]	Clinical data
[x]	[ ]	Dual use research of concern

Methods

n/a	Involved in the study	
[x]	[ ]	ChIP-seq
[x]	[ ]	Flow cytometry
[x]	[ ]	MRI-based neuroimaging

Antibodies

Antibodies used	1. anti-PAD1<br>2. anti-EP procyclin
Validation	anti-PAD1: For detection of Trypanosoma brucei PAD1 protein by immunofluorescence, mouse, previously validated Dean, S., Marchetti, R., Kirk, K. & Matthews, K. R. A surface transporter family conveys the trypanosome differentiation signal. Nature 459, 213–217 (2009).<br><br>anti-EP Procyclin: For detection of Trypanosoma brucei procyclin protein by immunofluorescence mouse, Ceder Lane Labs, CLP001AP, validated by manufacturer. "Cedarlane's Anti-Trypanosoma brucei procyclin Monoclonal Antibody recognizes the EP epeat portion of this molecule. This antibody can be used for ELISA's, immunoblotting and immunofluorescence on living and fixed parasites. The antibody does not cross-react with other species of trypanosomes or with Leishmania."

Eukaryotic cell lines

Policy information about cell lines

Cell line source(s)	EATRO 1125 AnTat1.1 90:13 and ZC3H20 KO null cell lines were provided by the Matthews Lab group.
Authentication	Cell lines were cultured in the antibiotics each is stated to be resistant to in order to authenticate prior to beginning experiments. PCR with specific primers to T brucei ZC3H20 genome region was used previously to verify ZC3H20 KO identify (DOI: 10.1016/j.cell.2018.10.041)
Mycoplasma contamination	No mycoplasma testing was performed
Commonly misidentified lines (See ICLAC register)	No commonly misidentified cell lines were used in this study
